# Evaluating the potential effect of PCSK9 inhibitors on the risk of sudden cardiac death and ventricular arrhythmias: A meta-analysis of randomized controlled trials

**DOI:** 10.1371/journal.pone.0329676

**Published:** 2025-08-08

**Authors:** Lei Zhang, Yuan-Yuan Li, Xue-Hui Liu, Hong-Jun Liu, Qiang Xu

**Affiliations:** 1 Department of Cardiology, Yichang Hospital of Traditional Chinese Medicine, Yichang, China; 2 Traditional Chinese Medicine Hospital of China Three Gorges University, China Three Gorges University, Yichang, China; 3 Chengdong Community Service Center of Wujiagang District, Yichang, China; Shaheed Rajaei Cardiovascular Medical and Research Center: Rajaie Cardiovascular Medical and Research Center, IRAN, ISLAMIC REPUBLIC OF

## Abstract

**Background:**

Proprotein convertase subtilisin/kexin type 9 (PCSK9) inhibitors are a new class of drugs used for the treatment of dyslipidemia. PCSK9 inhibitors have been shown to remarkably reduce cardiovascular events in patients at high risk, but data on their impact on sudden cardiac death (SCD) and ventricular arrhythmias are limited. This study aimed to evaluate whether PCSK9 inhibitor therapy reduces the risk of SCD and ventricular arrhythmias.

**Methods:**

PubMed and Embase were searched up to September 1, 2024 and combined with data from ClinicalTrials.gov. Randomized controlled trials of PCSK9 inhibitors with ≥ 450 patients and follow-up of ≥ 48 weeks were considered for inclusion. Primary outcomes were the incidence of SCD and ventricular arrhythmias. We used a random-effects model to synthesize the data, calculating risk ratio (RR) and 95% confidence intervals (CI). Heterogeneity between studies was assessed with *I*² statistics. Risk of bias was assessed using the Cochrane risk of bias tool.

**Results:**

A total of 12 articles with 16 trials involving 90,764 patients were included. The follow-up duration ranged from 48 weeks to 3.4 years. PCSK9 inhibitor therapy did not significantly reduce the risk of SCD (RR 0.83, 95% CI 0.54–1.28; P = 0.40; *I*^2^ = 0%), ventricular arrhythmias (RR 0.81, 95% CI 0.60–1.09; P = 0.17; *I*^2^ = 0%), and cardiac arrest (RR 1.20, 95% CI 0.61–2.33; P = 0.60; *I*^2^ = 0%).

**Conclusion:**

PCSK9 inhibitor therapy did not significantly reduce the risk of SCD and ventricular arrhythmias.

## Introduction

Sudden cardiac death (SCD) and ventricular arrhythmias are a major global health problem [1]. Despite major advancements in prevention, diagnostics, and medicine in the past decades, SCD remains a medical and societal challenge, accounting for approximately 50% of all cardiovascular deaths and 20% of total mortality worldwide [2,3]. The majority of SCD cases are attributed to ventricular tachyarrhythmias, which are frequently triggered by coronary artery disease (CAD), particularly acute coronary events, indicating that CAD is the most common underlying cause [2–4]. Elevated low-density lipoprotein cholesterol (LDL-C) is a well-established, independent risk factor for CAD [5], suggesting that lipid-lowering therapies may play a critical role in mitigating the risk of ventricular arrhythmias and SCD.

Previous studies have demonstrated that lowering LDL-C levels decreases the risk of ventricular arrhythmias and SCD [6–9]. Statins, the most widely used and well-tolerated lipid-lowering drugs, have shown modest benefits in reducing the risk of SCD [7]. Recently, PCSK9 inhibitors, a new class of lipid-lowering drugs, have emerged as highly effective therapies, capable of reducing LDL-C levels by approximately 60% [5]. Similar to statins, PCSK9 inhibitors significantly lower LDL-C levels and reduce the risk of major adverse cardiovascular events (MACE) [5,10]. Current guidelines recommend PCSK9 inhibitors for patients who fail to achieve LDL-C targets with the maximum tolerated dose of statins or for those who are statin-intolerant [11]. However, reliable evidence from randomized controlled trials (RCTs) regarding the effects of PCSK9 inhibitors on SCD and ventricular arrhythmias remains limited. Many large-scale trials have focused on non-cardiovascular endpoints and have not reported these specific outcomes.

To address this gap, we conducted a systematic review and meta-analysis of large RCTs to assess whether PCSK9 inhibitor therapy can reduce the risk of SCD and ventricular arrhythmias. This study aims to provide a comprehensive assessment of the potential effects of PCSK9 inhibitors in this area of cardiovascular health.

## Methods

Our study was conducted according to the Preferred Reporting Items for Systematic Reviews and Meta-Analyses (PRISMA) statement [12].

### Search strategy

We searched PubMed and Embase from their inception to September 1, 2024. Searched terms included “PCSK9”, “alirocumab”, “evolocumab”, “bococizumab” and “inclisiran”. The search was limited to RCT with no language restrictions. The full search strategy is presented in supporting information (S1 Table). Reference lists of included articles and relevant published meta-analyses were manually searched for additional eligible trials.

### Study selection

The initial search records were imported into EndNote software, where duplicate records were removed. Two authors (ZL and LYY) independently screened the remaining articles by title and abstract. For potentially eligible articles, full-text manuscripts were retrieved and assessed independently. There were no restrictions on participants’ characteristics or study endpoints. Studies were included if they met the following criteria: 1) RCT design; 2) sample size of ≥ 450 patients and follow-up duration of ≥ 48 weeks; 3) comparison of PCSK9 inhibitors with placebo or active treatments; and 4) reporting outcomes of interest, including SCD, ventricular tachycardia, ventricular fibrillation, or cardiac arrest. Trials with duplicate data were excluded; only the study with the larger sample size reporting the outcomes of interest was retained.

Two articles on bococizumab [13,14] also met the selection criteria. However, Pfizer discontinued the production of bococizumab due to its higher level of immunogenicity and higher rate of injection-site reactions [15]. To address potential selection bias from excluding these trials, we conducted a sensitivity analysis that included the bococizumab trials and reported the pooled outcomes separately.

### Data extraction

Two authors (ZL and LYY) independently extracted data by using a pre-specified data collection form. For each trial, we extracted the following information: first author, trial name, year of publication, participant characteristics, follow-up duration, PCSK9 inhibitor type and dose, NCT number, and the number of patients with any of the following events: sudden cardiac death, ventricular tachycardia or fibrillation, or cardiac arrest. If the manuscript did not report outcomes of interest, we searched the supplementary material and the adverse events from ClinicalTrials.gov. Disagreements were resolved by consensus.

### Assessment of risk of bias

Two authors (ZL and LYY) independently assessed the risk of bias using the Cochrane “Risk of bias” tool [16]. The following domains were evaluated: random sequence generation, allocation, blinding of participants and personnel, blinding of outcome assessment, incomplete outcome data, selective reporting, and other potential sources of bias. We graded individual items as having low, unclear, or high risk of bias. Discrepancies were resolved by consensus.

### Statistical analysis

The Mantel–Haenszel method was used to calculate pooled risk ratio (RR) with 95% confidence intervals (CI) for the outcomes of interest. Studies with no events in both arms were excluded from the meta-analysis [17]. A random-effects model was used regardless of heterogeneity. Statistical heterogeneity across trials was assessed by the *I*^2^ statistic, and *I*^2 ^> 50% indicated significant heterogeneity [18]. Pre-specified subgroup analyses focused on the type of PCSK9 inhibitor and the study endpoints (cardiovascular vs non-cardiovascular). To assess the consistency of outcomes, sensitivity analyses were performed by including SPIRE trials and excluding ORION trials (due to a different mechanism of action compared to PCSK9 inhibitors). Publication bias was evaluated by funnel plots. When each arm of a meta-analysis has at least ten events, a generalized linear mixed model was used to assess outcome robustness [17]. A two-sided P-value < 0.05 was considered statistically significant. The certainty of evidence for each outcome was evaluated using the Grading of Recommendation Assessment, Development and Evaluation (GRADE) framework. All analyses were conducted using Review Manager Software (RevMan version 5.4; The Nordic Cochrane Centre, Cochrane Collaboration), R statistical software (Version 4.4.1), and GRADEprofiler (Version 3.6).

## Results

### Trial selection

**Fig 1:**
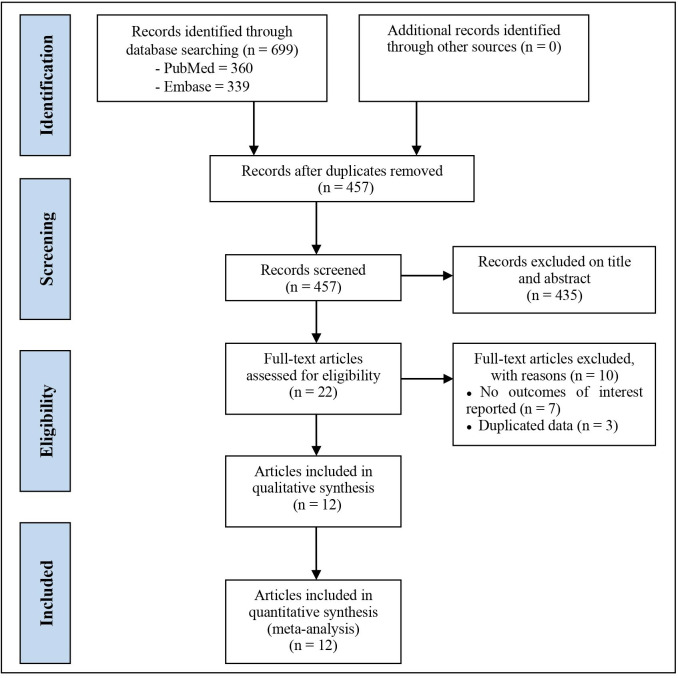
Study flow diagram.

The literature search yielded 699 articles, 242 were excluded for duplicates. Of these, 435 were removed by screening titles and abstracts. The full texts of the remaining 22 articles were read for further assessment. Finally, a total of 12 articles [13,14,19–28] with 16 trials involving 90,764 patients were included, of which 12 trials were selected in the primary analyses, and 4 trials were added in the sensitivity analyses. The search strategy is presented in Fig 1.

### Characteristics of included trials

The characteristics of the included trials are summarized in Table 1. Among the 12 trials included in the primary analysis, one trial compared a PCSK9 inhibitor with ezetimibe, nine trials compared PCSK9 inhibitors with placebo, and two trials compared PCSK9 inhibitor plus standard therapy with standard therapy alone. PCSK9 inhibitors used were alirocumab (5 studies, 23,274 patients), evolocumab (4 studies, 33,537 patients), bococizumab (4 studies, 30,323 patients), and inclisiran (3 studies, 3,660 patients). Fifteen trials reported ventricular arrhythmias, while five trials reported SCD. The trials were published between 2015 and 2020. The sample size ranged from 482 to 27,564. The mean age ranged from 51.9 to 66.1 years. The percentage of males ranged from 47.1% to 75.5%. The duration of follow-up ranged from 48 weeks to 3.4 years. The ODYSSEY OUTCOMES trial [26] enrolled patients with a recent acute coronary syndrome, while the remaining trials included patients with stable CVD, cardiovascular risk factors, or both. None of the 16 trials reported the outcomes of interest in their manuscripts.

**Table 1:** Baseline characteristics of the trials included.

Trial and NCT number	Main inclusion criteria	Total number of participants	Follow-up duration	Mean age (years)	Male (%)	MI (%)	Treatment		Background therapy	FH (%)
							Intervention	Control		
Cannon CP, 2015 [19] ODYSSEY COMBO Ⅱ NCT01644188	HC and CHD or CHD risk equivalent	720	52 weeks	61.6	73.6	60.0	ALI 75 mg Q2W or to 150 mg Q2W	Ezetimibe	Statin	NA
Robinson JG, 2015 [21] ODYSSEY LONG TERM, NCT01507831	HeFH/CHD/CHD risk equivalent	2,341	78 weeks	60.5	61.8	NA	ALI 150 mg Q2W	Placebo	SOC	17.7
Kastelein JJ, 2015 [20] ODYSSEY FH I, NCT01623115	HeFH	486	78 weeks	51.9	56.4	NA	ALI 75 mg/UP to 150 mg Q2W	Placebo	Statin and possible addition of other LLTs	100
Sabatine MS, 2015 [22] OSLER-1, NCT01439880	HC	1,324	52 weeks	58.0	50.8	9.4	EVO 420 mg Q4W + SOC	SOC	SOC	9.9
Sabatine MS, 2015 [22] OSLER-2, NCT01854918		3,681	48 weeks				EVO 140 mg Q2W or to 420 mg Q4W	SOC	SOC	
Nicholls SJ, 2016 [23] GLAGOV, NCT01813422	Angiographic coronary disease	968	76 weeks	59.8	72.2	35.1	EVO 420 mg Q4W	Placebo	Statin	NA
Roth EM, 2016 [24] ODYSSEY CHOICE 1, NCT01926782	HC with moderate to very High CVD risk	803	48 weeks	60.8	57.5	NA	ALI 75 mg Q2W or to 300 mg Q4W	Placebo	With or without statin	NA
Sabatine MS, 2017 [25] FOURIER, NCT01764633	ASCVD	27,564	157 weeks	62.5	75.5	81.1	EVO 140 mg Q2W or to 420 mg Q4W	Placebo	Statin	NA
Ridker PM, 2017 [13] SPIRE-1, NCT01975376	CVD/DM/CKD/PVD/FH	16,817	3.4 years	63.3	73.6	NA	BOC 150 mg Q2W	Placebo	Statin	1.8
Ridker PM, 2017 [13] SPIRE-2, NCT01975389		10,621	3.4 years	62.4	65.4	NA	BOC 150 mg Q2W	Placebo	Statin	7.3
Ridker PM, 2017 [14] SPIRE-LDL, NCT01968967	HC	2,139	58 weeks	62.0	59.4	NA	BOC 150 mg Q2W	Placebo	Statin	1.9
Ridker PM, 2017 [14] SPIRE-LL, NCT02100514	HC	746	58 weeks	61.6	55.8	NA	BOC 150 mg Q2W	Placebo	Statin	7.0
Schwartz GG, 2018 [26] ODYSSEY OUTCOMES, NCT01663402	ACS	18,924	134 weeks	58.6	74.8	83.0	ALI 75 mg Q2W	Placebo	Statin	NA
Raal FJ, 2020 [27] ORION-9 NCT03397121	HeFH	482	540 days	54.7	47.1	NA	INC 284 mg Day 1, Day 90, and then every 6 months	Placebo	Statin	100
Ray KK, 2020 [28] ORION-10, NCT03399370	ASCVD	1,561	540 days	66.1	69.4	NA	INC 284 mg Day 1, Day 90, and then every 6 months	Placebo	Statin	1.3
Ray KK, 2020 [28] ORION-11, NCT03400800	ASCVD or ASCVD risk equivalent	1,617	540 days	64.8	71.7	NA				1.7

ACS, acute coronary syndrome; ALI, alirocumab; ASCVD, atherosclerosis cardiovascular disease; BOC, bococizumab; NA, data not available; CHD, coronary heart disease; CKD, chronic kidney disease; CVD, cardiovascular disease; DM, diabetes mellitus; EVO, evolocumab; FH, familial hypercholesterolemia; HC, hypercholesterolaemia; HeFH, heterozygous familial hypercholesterolemia; INC, inclisiran; LLT, lipid-lowering therapy; PVD, peripheral vascular disease; Q2W, once two weeks; Q4W, once four weeks; SOC, standard of care.

### Risk of bias

**Fig 2:**
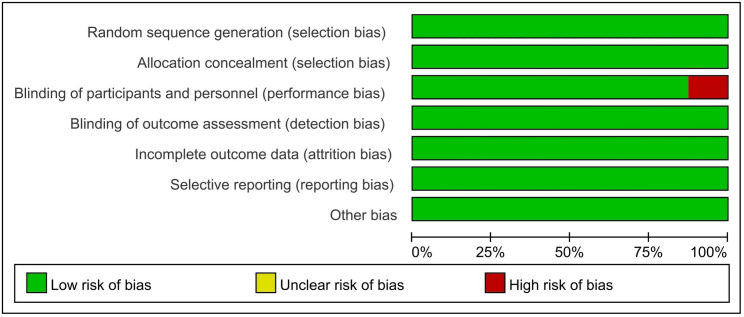
Risk of bias graph.

Details of risk of bias are summarized in the supporting information. Overall, two trials were at high risk of bias [22], the remaining fourteen trials were judged as at low risk of bias [13,14,19–21,23–28]. The risk of bias graph is shown in Fig 2.

### Sudden cardiac death

**Fig 3:**
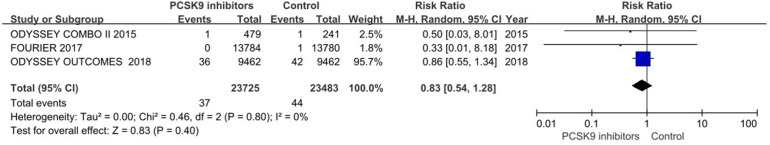
PCSK9 inhibitors and sudden cardiac death.

SCD was reported in five trials [13,19,25,26], involving a total of 74,523 patients. Among these, two trials [19,26] evaluated alirocumab, two trials [13] evaluated bococizumab, and one trial [25] evaluated evolocumab. The majority of SCD was reported in the ODYSSEY OUTCOMES trial [26]. There was no significant reduction in risk of SCD in patients using PCSK9 inhibitors as compared to controls but with a trend favoring treatment (RR 0.83, 95% CI 0.54–1.28; P = 0.40; Fig 3). A generalized linear mixed model showed a same outcome. Sensitivity analysis yielded similar results (RR 0.83, 95% CI 0.56–1.24; P = 0.37). No significant heterogeneity was observed across trials (*I*^2^ = 0%). Funnel plots were not performed to assess the publication bias because of the small number of trials included.

### Ventricular arrhythmias

**Fig 4:**
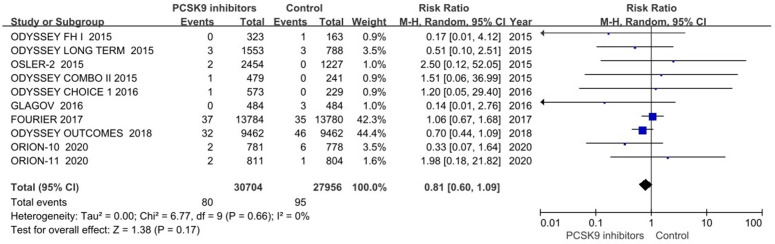
PCSK9 inhibitors and ventricular arrhythmias.

**Fig 5:**
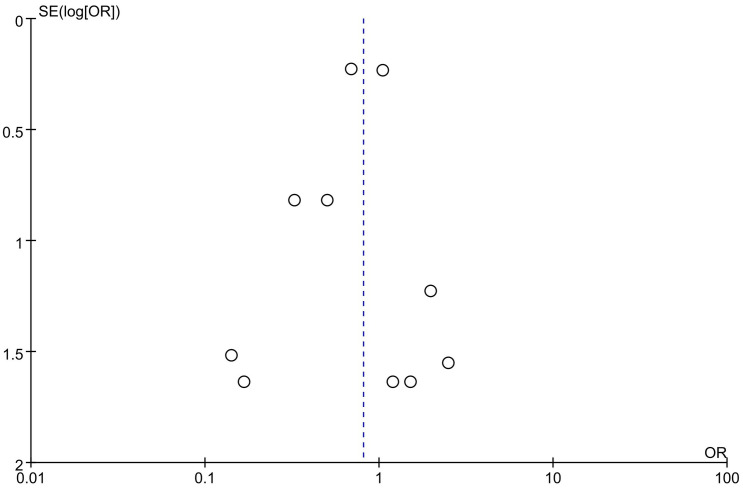
Funnel plot for ventricular arrhythmias.

Ventricular arrhythmias, defined as ventricular tachycardia or fibrillation, were reported in 15 trials [13,14,19–26,28]. Most events were reported in the FOURIER trial [25] and the ODYSSEY OUTCOMES trial [26]. OSLER-1 trial [22] was excluded from meta-analysis due to no events in either arm. In the primary analysis of 10 trials [19–26,28], PCSK9 inhibitors did not significantly reduce the risk of ventricular arrhythmias (RR 0.81, 95% CI 0.60–1.09; P = 0.17; *I*^2^ = 0%; Fig 4). However, there was a trend toward a lower incidence of ventricular arrhythmias in the treatment group compared to the controls. Funnel plot analysis suggested no significant publication bias (Fig 5). Additionally, a generalized linear mixed model also showed no significant risk reduction (RR 0.79, 95% CI 0.58–1.06; P = 0.12).

In subgroup analysis based on PCSK9 inhibitor type yielded results similar to overall analysis (alirocumab: RR 0.68, 95%CI 0.45–1.04, P = 0.07, *I*^2^ = 0%; evolocumab: RR 1.01, 95%CI 0.57–1.81, P = 0.98, *I*^2^ = 3%; inclisiran: RR 0.64, 95%CI 0.12–3.51, P = 0.22, *I*^2^ = 32%). The test for subgroup differences was nonsignificant (P = 0.55, *I*^2^ = 0%). Similarly, there were no significant differences between the comparison groups in the trials focusing on non-cardiovascular endpoints (RR 0.51, 95%CI 0.19–1.41, P = 0.20, *I*^2^ = 0%) or cardiovascular endpoints (RR 0.85, 95%CI 0.62–1.16, P = 0.30, *I*^2^ = 0%). The test for subgroup differences was nonsignificant (P = 0.35, *I*^2^ = 0%).

Sensitivity analyses, including SPIRE trials (RR 0.84, 95%CI 0.64–1.10; P = 0.21, *I*^2^ = 0%) or excluding ORION trials (RR 0.82, 95%CI 0.61–1.12; P = 0.22, *I*^2^ = 0%), showed consistent outcomes.

### Cardiac arrest

**Fig 6:**
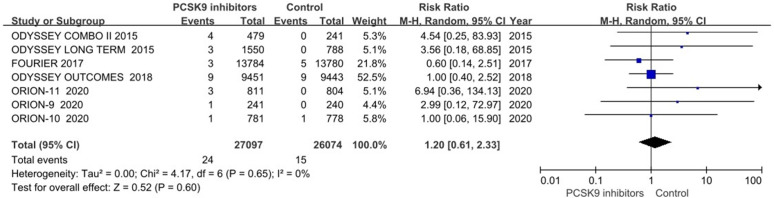
PCSK9 inhibitors and cardiac arrest.

Cardiac arrest was reported in 12 trials [13,14,19,21,22,25–28]. The OSLER-1 and OSLER-2 trials [22], which reported no events in both arms, were excluded from meta-analysis. In the primary analysis of seven trials, PCSK9 inhibitors therapy did not significantly reduce the risk of cardiac arrest (RR 1.20, 95%CI 0.61–2.33; P = 0.60; *I*^2^ = 0%; Fig 6). A generalized linear mixed model also showed no significant risk reduction (RR 1.47, 95% CI 0.77–2.80; P = 0.25). The overall effects were consistent in sensitivity analyses including SPIRE trials (RR 1.33, 95%CI 0.78–2.28; P = 0.29) or excluding ORION trials (RR 1.04, 95%CI 0.50–2.15; P = 0.92).

### Certainty of evidence

GRADE certainty of evidence for the outcomes is shown in supporting information (S5 Table). The GRADE level of evidence is low for SCD, ventricular arrhythmias, and cardiac arrest.

## Discussion

To our knowledge, this is the first meta-analysis to evaluate the potential effect of PCSK9 inhibitors therapy on the risk of SCD and ventricular arrhythmias using data from RCTs. We found no significant differences in the incidence of SCD and ventricular arrhythmias between the PCSK9 inhibitors and control group. The results of pre-defined subgroup and sensitivity analyses appeared to be consistent with the primary analysis; however, there was a trend suggesting a potential antiarrhythmic effect of long-term PCSK9 inhibitor therapy.

The majority of SCD are caused by ventricular arrhythmias, often triggered by acute coronary events [29]. LDL-C is a primary cause of atherosclerotic cardiovascular disease (ASCVD). Lipid-lowering therapies have been shown to reduce ASCVD and all-cause mortality [5]. It is plausible that the beneficial effects of lipid-lowering therapy may be attributed to the reduction of ventricular arrhythmias and SCD. The AVID trial demonstrated that lipid-lowering therapy reduced the recurrence of ventricular tachyarrhythmia in patients with atherosclerotic heart disease and implantable cardioverter defibrillator (ICD), suggesting an antiarrhythmic effect [6]. A meta-analysis of RCTs showed that statins therapy was associated with a significant 10% reduction in SCD [7]. Therefore, it is hypothesized that lipid-lowering therapy has direct or indirect antiarrhythmic effects.

PCSK9 inhibitors are powerful LDL-C lowering drugs, and their combination with statins has been shown to further reduce LDL-C levels and MACE [5]. Therefore, PCSK9 inhibitor therapy may reduce the incidence of ventricular arrhythmias or SCD. In our meta-analysis, the majority of the events of interest were reported in the FOURIER and ODYSSEY OUTCOMES trials. The FOURIER trial [25] demonstrated that evolocumab, when combined with statins, significantly reduced LDL-C levels and the risk of CVD events. Similarly, the ODYSSEY OUTCOMES trial [26], which evaluated alirocumab in patients with acute coronary syndrome receiving maximal statin ± ezetimibe over a median follow-up of 2.8 years, demonstrated a 15% relative risk reduction in composite ASCVD events. However, the pooled analysis of these two RCTs did not show a significant reduction in the risk of SCD or ventricular arrhythmias with PCSK9 inhibitors therapy, although a trend favoring treatment was observed. This may be attributed to the implementation of guideline-directed management and therapy, including the use of antiplatelet drugs, heart failure medications, revascularization techniques and ICD, all of which independently contribute to reducing the risk of SCD and arrhythmic events [3]. In addition, most of the included trials enrolled stable cardiovascular disease patients with relatively low SCD risk, which may have attenuated the potential antiarrhythmic effects of PCSK9 inhibitors therapy.

**Fig 7:**
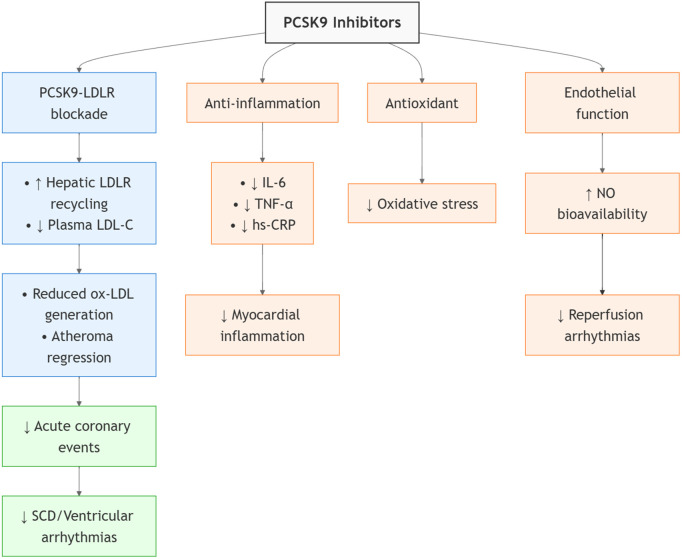
Potential mechanisms of PCSK9 inhibitor in SCD and ventricular arrhythmias. hs-CRP, high sensitivity C-reactive protein; LDLR, low-density lipoprotein receptor; IL-6, interleukin-6; SCD, sudden cardiac death; TNF-α, tumor necrosis factor-α.

PCSK9 inhibitor may exert beneficial effects on SCD and ventricular arrhythmias (Fig 7). First, SCD is most commonly associated with CAD, either as its initial manifestation or during the post-acute myocardial infarction period [30]. PCSK9 inhibitors bind to circulating PCSK9, preventing LDL receptor (LDLR) degradation and enhancing hepatic LDLR recycling, which lowers plasma LDL-C by 50−60% [5,10]. This results in reduced oxidized LDL generation, atheroma regression, and lower coronary events [23,26,31]. Additionally, improved coronary blood flow may reduce ischemic burden and decrease the risk of ventricular arrhythmias. In a rat model of ischemia/reperfusion injury, PCSK9 inhibitor was shown to reduce cardiac arrhythmias, suggesting potential cardioprotective effects beyond lipid-lowering [32]. Second, both experimental and clinical studies suggest that inflammation and oxidative stress play critical roles in cardiovascular pathology and increase the risk of life-threatening arrhythmias [33]. Treatment with PCSK9 inhibitor has been demonstrated a marked decrease in the levels of oxidative stress [34]. Ji J *et al* found that high sensitivity C-reactive protein (hs-CRP), tumor necrosis factor-α (TNF-α), and interleukin-6 (IL-6) levels were significantly lower in the PCSK9 inhibitor group than in the control group after treatment [35]. However, a meta-analysis of ten RCTs showed that short-term PCSK9 inhibitor therapy did not reduce hs-CRP levels, irrespective of the type of PCSK9 inhibitor and patient characteristics [36]. Third, PCSK9 inhibitors have been demonstrated to protect coronary artery endothelial function [37,38], which may decrease the probability of plaque rupture and prevent ischemia-induced electrophysiological disturbances that predispose patients to ventricular arrhythmias [6].

There are several limitations in our meta-analysis. First, the outcomes of interest in all included trials were reported as serious adverse events, rather than as pre-specified endpoints. Therefore, it is possible that the number of SCD or ventricular arrhythmias may have been underreported. Second, the precise incidence of SCD is difficult to ascertain due to its diverse definition and complex mechanisms. SCD accounts for approximately 50% of all cardiovascular deaths [3]. A total of 491 patients in the FOURIER trial and 511 patients in the ODYSSEY OUTCOMES trial died from cardiovascular causes. However, these trials only reported 79 events, accounting for 7.9% of all cardiovascular deaths, which is significantly lower than the average incidence of SCD. Third, there were no standardized definitions for ventricular arrhythmias in each trial, which may lead to reporting bias. Finally, the number of SCD and ventricular arrhythmias in each trial was relatively small, resulting in wide CIs. This indicates that the results may have been influenced by low statistical power.

## Conclusion

PCSK9 inhibitors did not significantly reduce the risk of SCD or ventricular arrhythmias, though a non-significant trend suggested potential benefits for patients with ASCVD or higher cardiovascular risk. This meta-analysis should be considered hypothesis-generating due to reliance on adverse event reporting rather than pre-defined endpoints.

## Supporting information

S1 Table The full search strategy. (DOCX)

S2 Table All studies identified in the literature search. (XLSX)

S3 Table All data extracted from included studies. (DOCX)

S4 Table PRISMA 2020 checklist. (DOCX)

S5 Table GRADE certainty of evidence. (DOCX)

S1 File Additional sensitivity analysis. (DOCX)

S2 File Meta-analysis using Bayesian method. (DOCX)

## References

[1] Könemann H, Dagres N, Merino JL, Sticherling C, Zeppenfeld K, Tfelt-Hansen J, et al. Spotlight on the 2022 ESC guideline management of ventricular arrhythmias and prevention of sudden cardiac death: 10 novel key aspects. Europace. 2023;25(5):euad091. doi: 10.1093/europace/euad091 37102266 PMC10228619

[2] Marijon E, Narayanan K, Smith K, Barra S, Basso C, Blom MT, et al. The Lancet Commission to reduce the global burden of sudden cardiac death: a call for multidisciplinary action. Lancet. 2023;402(10405):883–936. doi: 10.1016/S0140-6736(23)00875-9 37647926

[3] Zeppenfeld K, Tfelt-Hansen J, de Riva M, Winkel BG, Behr ER, Blom NA, et al. 2022 ESC Guidelines for the management of patients with ventricular arrhythmias and the prevention of sudden cardiac death. Eur Heart J. 2022;43(40):3997–4126. doi: 10.1093/eurheartj/ehac262 36017572

[4] Huikuri HV, Castellanos A, Myerburg RJ. Sudden death due to cardiac arrhythmias. N Engl J Med. 2001;345(20):1473–82. doi: 10.1056/NEJMra000650 11794197

[5] Grundy SM, Stone NJ, Bailey AL, Beam C, Birtcher KK, Blumenthal RS, et al. 2018 AHA/ACC/AACVPR/AAPA/ABC/ACPM/ADA/AGS/APhA/ASPC/NLA/PCNA Guideline on the Management of Blood Cholesterol: A Report of the American College of Cardiology/American Heart Association Task Force on Clinical Practice Guidelines. Circulation. 2019;139(25):e1082–143. doi: 10.1161/CIR.0000000000000625 30586774 PMC7403606

[6] Mitchell LB, Powell JL, Gillis AM, Kehl V, Hallstrom AP, AVID Investigators. Are lipid-lowering drugs also antiarrhythmic drugs? An analysis of the Antiarrhythmics versus Implantable Defibrillators (AVID) trial. J Am Coll Cardiol. 2003;42(1):81–7. doi: 10.1016/s0735-1097(03)00498-4 12849664

[7] Rahimi K, Majoni W, Merhi A, Emberson J. Effect of statins on ventricular tachyarrhythmia, cardiac arrest, and sudden cardiac death: a meta-analysis of published and unpublished evidence from randomized trials. Eur Heart J. 2012;33(13):1571–81. doi: 10.1093/eurheartj/ehs005 22307462

[8] De Sutter J, Firsovaite V, Tavernier R. Prevention of sudden death in patients with coronary artery disease: do lipid-lowering drugs play a role?. Prev Cardiol. 2002;5(4):177–82. doi: 10.1111/j.1520.037x.2002.00731.x 12417826

[9] Tamargo J, Caballero R, Gómez R, Núñez L, Vaquero M, Delpón E. Lipid-lowering therapy with statins, a new approach to antiarrhythmic therapy. Pharmacol Ther. 2007;114(1):107–26. doi: 10.1016/j.pharmthera.2006.12.002 17287023

[10] Sabatine MS. PCSK9 inhibitors: clinical evidence and implementation. Nat Rev Cardiol. 2019;16(3):155–65. doi: 10.1038/s41569-018-0107-8 30420622

[11] Mach F, Baigent C, Catapano AL, Koskinas KC, Casula M, Badimon L, et al. 2019 ESC/EAS Guidelines for the management of dyslipidaemias: lipid modification to reduce cardiovascular risk. Eur Heart J. 2020;41(1):111–88. doi: 10.1093/eurheartj/ehz455 31504418

[12] Page MJ, McKenzie JE, Bossuyt PM, Boutron I, Hoffmann TC, Mulrow CD, et al. The PRISMA 2020 statement: an updated guideline for reporting systematic reviews. BMJ. 2021;372:n71. doi: 10.1136/bmj.n71 33782057 PMC8005924

[13] Ridker PM, Revkin J, Amarenco P, Brunell R, Curto M, Civeira F, et al. Cardiovascular Efficacy and Safety of Bococizumab in High-Risk Patients. N Engl J Med. 2017;376(16):1527–39. doi: 10.1056/NEJMoa1701488 28304242

[14] Ridker PM, Tardif J-C, Amarenco P, Duggan W, Glynn RJ, Jukema JW, et al. Lipid-Reduction Variability and Antidrug-Antibody Formation with Bococizumab. N Engl J Med. 2017;376(16):1517–26. doi: 10.1056/NEJMoa1614062 28304227

[15] Ferri N, Corsini A, Sirtori CR, Ruscica M. Bococizumab for the treatment of hypercholesterolaemia. Expert Opin Biol Ther. 2017;17(2):237–43. doi: 10.1080/14712598.2017.1279602 28060539

[16] Higgins JP, Savović J, Page MJ, Elbers RG, Sterne JA. Assessing risk of bias in a randomized trial. Cochrane handbook for systematic reviews of interventions. 2019. p. 205–28.

[17] Xu C, Zhou X, Zorzela L, Ju K, Furuya-Kanamori L, Lin L, et al. Utilization of the evidence from studies with no events in meta-analyses of adverse events: an empirical investigation. BMC Med. 2021;19(1):141. doi: 10.1186/s12916-021-02008-2 34126999 PMC8204528

[18] Higgins JPT, Thompson SG. Quantifying heterogeneity in a meta-analysis. Stat Med. 2002;21(11):1539–58. doi: 10.1002/sim.1186 12111919

[19] Cannon CP, Cariou B, Blom D, McKenney JM, Lorenzato C, Pordy R, et al. Efficacy and safety of alirocumab in high cardiovascular risk patients with inadequately controlled hypercholesterolaemia on maximally tolerated doses of statins: the ODYSSEY COMBO II randomized controlled trial. Eur Heart J. 2015;36(19):1186–94. doi: 10.1093/eurheartj/ehv028 25687353 PMC4430683

[20] Kastelein JJP, Ginsberg HN, Langslet G, Hovingh GK, Ceska R, Dufour R, et al. ODYSSEY FH I and FH II: 78 week results with alirocumab treatment in 735 patients with heterozygous familial hypercholesterolaemia. Eur Heart J. 2015;36(43):2996–3003. doi: 10.1093/eurheartj/ehv370 26330422 PMC4644253

[21] Robinson JG, Farnier M, Krempf M, Bergeron J, Luc G, Averna M, et al. Efficacy and safety of alirocumab in reducing lipids and cardiovascular events. N Engl J Med. 2015;372(16):1489–99. doi: 10.1056/NEJMoa1501031 25773378

[22] Sabatine MS, Giugliano RP, Wiviott SD, Raal FJ, Blom DJ, Robinson J, et al. Efficacy and safety of evolocumab in reducing lipids and cardiovascular events. N Engl J Med. 2015;372(16):1500–9. doi: 10.1056/NEJMoa1500858 25773607

[23] Nicholls SJ, Puri R, Anderson T, Ballantyne CM, Cho L, Kastelein JJP, et al. Effect of Evolocumab on Progression of Coronary Disease in Statin-Treated Patients: The GLAGOV Randomized Clinical Trial. JAMA. 2016;316(22):2373–84. doi: 10.1001/jama.2016.16951 27846344

[24] Roth EM, Moriarty PM, Bergeron J, Langslet G, Manvelian G, Zhao J, et al. A phase III randomized trial evaluating alirocumab 300 mg every 4 weeks as monotherapy or add-on to statin: ODYSSEY CHOICE I. Atherosclerosis. 2016;254:254–62. doi: 10.1016/j.atherosclerosis.2016.08.043 27639753

[25] Sabatine MS, Giugliano RP, Keech AC, Honarpour N, Wiviott SD, Murphy SA, et al. Evolocumab and Clinical Outcomes in Patients with Cardiovascular Disease. N Engl J Med. 2017;376(18):1713–22. doi: 10.1056/NEJMoa1615664 28304224

[26] Schwartz GG, Steg PG, Szarek M, Bhatt DL, Bittner VA, Diaz R, et al. Alirocumab and Cardiovascular Outcomes after Acute Coronary Syndrome. N Engl J Med. 2018;379(22):2097–107. doi: 10.1056/NEJMoa1801174 30403574

[27] Raal FJ, Kallend D, Ray KK, Turner T, Koenig W, Wright RS, et al. Inclisiran for the Treatment of Heterozygous Familial Hypercholesterolemia. N Engl J Med. 2020;382(16):1520–30. doi: 10.1056/NEJMoa1913805 32197277

[28] Ray KK, Wright RS, Kallend D, Koenig W, Leiter LA, Raal FJ, et al. Two Phase 3 Trials of Inclisiran in Patients with Elevated LDL Cholesterol. N Engl J Med. 2020;382(16):1507–19. doi: 10.1056/NEJMoa1912387 32187462

[29] Shomanova Z, Ohnewein B, Schernthaner C, Höfer K, Pogoda CA, Frommeyer G, et al. Classic and Novel Biomarkers as Potential Predictors of Ventricular Arrhythmias and Sudden Cardiac Death. J Clin Med. 2020;9(2):578. doi: 10.3390/jcm9020578 32093244 PMC7074455

[30] Sara JD, Eleid MF, Gulati R, Holmes DR Jr. Sudden cardiac death from the perspective of coronary artery disease. Mayo Clin Proc. 2014;89(12):1685–98. doi: 10.1016/j.mayocp.2014.08.022 25440727

[31] Carnevale R, Bartimoccia S, Nocella C, Di Santo S, Loffredo L, Illuminati G, et al. LDL oxidation by platelets propagates platelet activation via an oxidative stress-mediated mechanism. Atherosclerosis. 2014;237(1):108–16. doi: 10.1016/j.atherosclerosis.2014.08.041 25238217

[32] Palee S, McSweeney CM, Maneechote C, Moisescu DM, Jaiwongkam T, Kerdphoo S, et al. PCSK9 inhibitor improves cardiac function and reduces infarct size in rats with ischaemia/reperfusion injury: Benefits beyond lipid-lowering effects. J Cell Mol Med. 2019;23(11):7310–9. doi: 10.1111/jcmm.14586 31557388 PMC6815840

[33] Andelova K, Bacova BS, Sykora M, Hlivak P, Barancik M, Tribulova N. Mechanisms Underlying Antiarrhythmic Properties of Cardioprotective Agents Impacting Inflammation and Oxidative Stress. Int J Mol Sci. 2022;23(3):1416. doi: 10.3390/ijms23031416 35163340 PMC8835881

[34] Cammisotto V, Baratta F, Castellani V, Bartimoccia S, Nocella C, D’Erasmo L, et al. Proprotein Convertase Subtilisin Kexin Type 9 Inhibitors Reduce Platelet Activation Modulating ox-LDL Pathways. Int J Mol Sci. 2021;22(13):7193. doi: 10.3390/ijms22137193 34281247 PMC8267646

[35] Ji J, Wei X, Chen W, Wan D, Han W, Liu H. Effects of early PCSK9 inhibitor application on inflammation levels and microcirculatory function after PCI in patients with NSTE-ACS. Am J Transl Res. 2023;15(5):3586–96. 37303640 PMC10251011

[36] Cao Y-X, Li S, Liu H-H, Li J-J. Impact of PCSK9 monoclonal antibodies on circulating hs-CRP levels: a systematic review and meta-analysis of randomised controlled trials. BMJ Open. 2018;8(9):e022348. doi: 10.1136/bmjopen-2018-022348 30287608 PMC6173233

[37] Leucker TM, Gerstenblith G, Schär M, Brown TT, Jones SR, Afework Y, et al. Evolocumab, a PCSK9-Monoclonal Antibody, Rapidly Reverses Coronary Artery Endothelial Dysfunction in People Living With HIV and People With Dyslipidemia. J Am Heart Assoc. 2020;9(14):e016263. doi: 10.1161/JAHA.120.016263 32674634 PMC7660736

[38] Ziogos E, Chelko SP, Harb T, Engel M, Vavuranakis MA, Landim-Vieira M, et al. Platelet activation and endothelial dysfunction biomarkers in acute coronary syndrome: the impact of PCSK9 inhibition. Eur Heart J Cardiovasc Pharmacother. 2023;9(7):636–46. doi: 10.1093/ehjcvp/pvad051 37468450 PMC12098939

