# Supplementary material for: Evaluating the potential effect of PCSK9 inhibitors on the risk of sudden cardiac death and ventricular arrhythmias: A meta-analysis of randomized controlled trials
Source: PLoS One. 2025 Aug 8;20(8):e0329676. doi: 10.1371/journal.pone.0329676 (PMC12334025; doi:10.1371/journal.pone.0329676)
Supplement: S1 Table — (DOCX) [file pone.0329676.s001.docx]

**S1 Table.** The full search strategy.

| #1 | PCSK9 [ti/ab] |
| --- | --- |
| #2 | alirocumab [ti/ab] |
| #3 | evolocumab [ti/ab] |
| #4 | bococizumab [ti/ab] |
| #5 | inclisiran [ti/ab] |
| #6 | #1 OR #2 OR #3 OR #4 OR #5 |
| #7 | randomized controlled trial [pt] |
| #8 | #6 AND #7 |
