# Supplementary material for: Evaluating the potential effect of PCSK9 inhibitors on the risk of sudden cardiac death and ventricular arrhythmias: A meta-analysis of randomized controlled trials
Source: PLoS One. 2025 Aug 8;20(8):e0329676. doi: 10.1371/journal.pone.0329676 (PMC12334025; doi:10.1371/journal.pone.0329676)
Supplement: S3 Table — (DOCX) [file pone.0329676.s003.docx]

**S3 Table.** All data extracted from included studies.

**Zhang L and Li YY extracted all data in September 2024.**

**Title**: Efficacy and safety of alirocumab in high cardiovascular risk patients with inadequately controlled hypercholesterolaemia on maximally tolerated doses of statins: the ODYSSEY COMBO II randomized controlled trial.

| Year of publication: 2015 | | |
| --- | --- | --- |
| First author: Cannon CP | | |
| NCT: NCT01644188 | | |
| **Methods** Type of RCT: A double-blind, double-dummy, active-controlled, parallel-group, 104-week  study  Settings: Outpatient care  Duration: 104 weeks | | |
| **Participants** Number of participants: 720  Male: 530 (73.6%)  Mean age: 61.6 years  Participants with FH: no data  History of CVD: 649 (MI 431)  Inclusion criteria: Participants with hypercholesterolemia and established coronary heart disease (CHD) or CHD risk equivalents who were not adequately controlled with a maximally tolerated daily dose of statin at stable dose for at least 4 weeks prior to the screening visit (Week -2).  Exclusion criteria   - Age < 18 or legal age of adulthood, whichever was greater - Participants without established CHD or CHD risk equivalents - LDL-C <70 mg/dL (<1.81 mmol/L) and participants with a history of documented cardiovascular disease - LDL-C <100 mg/dL (<2.59 mmol/L) and participants without a history of documented CV disease - Fasting serum triglycerides >400 mg/dL (>4.52 mmol/L) | | |
| **Interventions** Background therapy: A maximally tolerated dose of statin therapy  Randomized therapy: Patients were randomized to either alirocumab 75 mg every 2 weeks (plus oral placebo for ezetimibe daily) or 10 mg oral ezetimibe daily (plus placebo Q2W for alirocumab). The dose in the alirocumab arm (only) was automatically increased at Week 12 to 150 mg Q2W if the Week-8 LDL-C value was ≥1.8 mmol/L.  Alirocumab dose: 75 mg Q2W or 150mg Q2W | | |
| **Primary endpoints**: The primary endpoint was percent change in calculated LDL-C from baseline to Week 24, using all LDL-C values from Week 24 regardless of adherence to treatment. | | |
| **Outcomes of interest (PCSK9 inhibitor vs control)** VF: 1/479 vs 0/241  SCD: 1/479 vs 1/241  Cardiac arrest: 4/479 vs 0/241 | | |
| **Risk of bias** | | |
| **Bias** | **Authors’**  **judgement** | **Support for judgement** |
| Random sequence generation | Low | Used interactive voice response system (ALMAC company). |
| Allocation concealment | Low | Used a permuted-block design. |
| Blinding of participants and personnel | Low | Double-blind. |
| Blinding of outcome assessment | Low | Safety was assessed by analysing adverse-event reports and laboratory analyses. Laboratory analyses for all safety parameters were performed by a central laboratory. |
| Incomplete outcome data | Low | All participants were included in the adverse event analyses (52 weeks). |
| Selective reporting | Low | Safety analyses used a pre-specified cut-off corresponding to the last patient visit at Week 52 and included all data collected between 52 and 104 weeks. |
| Other bias | Low | No concerns outside the assessed risk of bias domains. |

**Title**: ODYSSEY FH I and FH II: 78 week results with alirocumab treatment in 735 patients with heterozygous familial hypercholesterolaemia.

| Year of publication: 2015 | | |
| --- | --- | --- |
| First author: Kastelein JJ | | |
| NCT: NCT01623115 | | |
| **Methods** Type of RCT: A randomized, double-blind, placebo-controlled Phase 3 studies.  Settings: Outpatient care  Duration: 78 weeks | | |
| **Participants** Number of participants: 486  Male: 274 (56.4%)  Mean age: 51.9 years  Participants with FH: 486  History of CVD: 225 (46%)  Inclusion criteria: Participants with heterozygous familial hypercholesterolemia who were not adequately controlled with their lipid-modifying therapy.  Exclusion criteria   - Age < 18 years or legal age of adulthood, whichever is greater - LDL-C < 70 mg/dL (1.81 mmol/L) and with cardiovascular disease - LDL-C < 100 mg/dL (2.59 mmol/L) and without cardiovascular disease - Fasting serum triglycerides > 400 mg/dL (4.52 mmol/L) - Known history of homozygous familial hypercholesterolemia | | |
| **Interventions** Background therapy: All patients were receiving stable high dose statin therapy, with or without other lipid-lowering therapy, for at least 4 weeks prior to screening.  Randomized therapy: Alirocumab vs placebo.  Alirocumab dose: Alirocumab 75 mg Q2W on top of stable lipid modifying therapy for 78 weeks. Alirocumab dose up-titrated to 150 mg from Week 12 when LDL-C levels ≥ 70 mg/dL (1.81 mmol/L) at Week 8. | | |
| **Primary endpoints**: The primary endpoint was the percent change in calculated LDL-C from baseline to Week 24. | | |
| **Outcomes of interest (PCSK9 inhibitor vs control)** VF: 0/323 vs 1/163 | | |
| **Risk of bias** | | |
| **Bias** | **Authors’**  **judgement** | **Support for judgement** |
| Random sequence generation | Low | Used interactive voice-response system or interactive web-response system. |
| Allocation concealment | Low | Randomization was stratified by history of MI or ischemic stroke, statin treatment. |
| Blinding of participants and personnel | Low | Double-blind |
| Blinding of outcome assessment | Low | All lipid measurements and laboratory tests were performed using standard procedures by a central laboratory. |
| Incomplete outcome data | Low | 85.8% of alirocumab-treated patients (87.7% of placebo) received study treatment for ≥76 weeks. All patients were included in safety analysis. |
| Selective reporting | Low | Safety was assessed through adverse events reports, laboratory data, electrocardiogram, and vital signs. |
| Other bias | Low | No concerns outside the assessed risk of bias domains. |

**Title**: Efficacy and safety of alirocumab in reducing lipids and cardiovascular events. (ODYSSEY LONG TERM)

| Year of publication: 2015 | | |
| --- | --- | --- |
| First author: Robinson JG | | |
| NCT: NCT01507831 | | |
| **Methods** Type of RCT: A phase 3, randomized, double-blind, placebo-controlled, parallel-group, multinational study  Settings: outpatient care  Duration: 78 weeks | | |
| **Participants** Number of participants: 2341  Male: 1457 (62.2%)  Mean age: 60.5 years  Participants with FH: 415 (17.7%)  History of CVD: 1607 (68.6%)  Inclusion criteria: Either A or B below and who were not adequately controlled with their lipid-modifying therapy:  A) Participants with heterozygous familial hypercholesterolemia (heFH) with or without established CHD or CHD risk equivalents OR  B) Participants with hypercholesterolemia together with established CHD or CHD risk equivalents.  Exclusion criteria   - Age < 18 years - LDL-C <70 mg/dL (< 1.81 mmol/L) - Fasting serum triglycerides > 400 mg/dL (>4.52 mmol/L) | | |
| **Interventions** Background therapy: All patients were required to be receiving either high-dose statin therapy or statin therapy at the maximum tolerated dose, with or without other lipid-lowering therapy, for at least 4 weeks before screening.  Randomized therapy: Alirocumab vs placebo.  Alirocumab dose: Alirocumab 150 mg Q2W added to stable LMT for 78 weeks. | | |
| **Primary endpoints**: The primary efficacy end point was the percentage change in calculated LDL cholesterol level from baseline to week 24, analyzed with the use of an intention-to-treat approach. | | |
| **Outcomes of interest (PCSK9 inhibitor vs control)** VT: 2/1553 vs 1/788  VF: 1/1553 vs 2/788  SCD: 36/1553 vs 42/788  Cardiac arrest: 9/1553 vs 9/788 | | |
| **Risk of bias** | | |
| **Bias** | **Authors’**  **judgement** | **Support for judgement** |
| Random sequence generation | Low | The randomized list of treatment kit numbers was computer generated by Sanofi and provided to a central allocation system. |
| Allocation concealment | Low | Study patients, investigators and study site personnel remained blinded to treatment allocation until after completion of the trial. |
| Blinding of participants and personnel | Low | Study patients, investigators and study site personnel remained blinded to treatment allocation until after completion of the trial. |
| Blinding of outcome assessment | Low | Safety end points were adverse events, including symptoms, laboratory abnormalities, vital-sign abnormalities, electrocardiographic abnormalities, and adjudicated cardiovascular events. The safety was monitored by safety monitoring committee. |
| Incomplete outcome data | Low | A total of 99.8% of the patients were included in the safety analysis. |
| Selective reporting | Low | Safety end points were adverse events, including symptoms, laboratory abnormalities, vital-sign abnormalities, electrocardiographic abnormalities, and adjudicated cardiovascular events. |
| Other bias | Low | No concerns outside the assessed risk of bias domains. |

**Title**: Efficacy and safety of evolocumab in reducing lipids and cardiovascular events. (OSLER)

| Year of publication: 2015 | | |
| --- | --- | --- |
| First author: Sabatine MS | | |
| NCT: NCT01439880 (OSLER-1), NCT01854918 (OSLER-2) | | |
| **Methods** Type of RCT: Two open-label, randomized, controlled studies  Settings: outpatient care  Duration: 52 weeks (OSLER-1), 48 weeks (OSLER-2) | | |
| **Participants** Male: 2255 (50.8%)  Mean age: 58.0 years  Participants with FH: 440  History of CVD: 1170 (MI 417)  **OSLER-1**  Number of participants: 1324  Inclusion criteria: Complete a qualifying evolocumab (AMG 145) parent study protocol, including: 20101154 (NCT01375777), 20101155 (NCT01380730), 20090158 (NCT01375751), 20090159 (NCT01375764), and 20110231 (NCT01652703).  Exclusion criteria   - Experienced a treatment-related serious adverse event that led to investigational product (IP) discontinuation in the parent study - Have an unstable medical condition, in the judgment of the investigator - Known sensitivity to any of the products to be administered during dosing - Currently enrolled in another investigational device or drug study (excluding evolocumab (AMG 145) parent study), or less than 30 days since ending another investigational device or drug study(s), or receiving other investigational agent(s)   **OSLER-2**  Number of participants: 3141  Inclusion criteria: Complete a qualifying evolocumab (AMG 145) parent study (ie, Study 20110114 [NCT01763827], 20110115 [NCT01763866], 20110116 [NCT01763905], 20110117 [NCT01763918], 20110109 [NCT01516879], 20120122 [NCT01953328], 20120332 [NCT01984424], 20120348 [NCT01849497], or 20120356 [NCT01879319]).  Exclusion criteria   - Experienced a treatment-related serious adverse event that led to study drug discontinuation in the parent study - Have an unstable medical condition, in the judgment of the investigator - Known sensitivity to any of the products to be administered during dosing - Currently enrolled in another investigational device or drug study (excluding evolocumab (AMG 145) parent study), or less than 30 days since ending another investigational device or drug study(s), or receiving other investigational agent(s) | | |
| **Interventions** Background therapy: Standard-of-care.  Randomized therapy: Evolocumab plus standard therapy vs standard therapy.  Evolocumab dose: Evolocumab was administered subcutaneously at a dose of 420 mg once a month in OSLER-1 and, on the basis of patient choice, at a dose of either 140 mg every 2 weeks or 420 mg once a month in OSLER-2. | | |
| **Primary endpoints**: The primary end point in the two trials was the incidence of adverse events. Additional safety end points included serious adverse events, adverse events leading to the discontinuation of the study drug (for patients in the evolocumab group), abnormalities in creatine kinase levels and liverfunction testing, and the development of binding and neutralizing antibodies against evolocumab. | | |
| **Outcomes of interest (PCSK9 inhibitor vs control)** **OSLER-1**  VT: 0/882 vs 0/442  Cardiac arrest: 0/882 vs 0/442  **OSLER-2**  VT: 2/2454 vs 0/1227  VF: 0/2454 vs 0/1227  Cardiac arrest: 0/2454 vs 0/1227 | | |
| **Risk of bias** | | |
| **Bias** | **Authors’**  **judgement** | **Support for judgement** |
| Random sequence generation | Low | Used an interactive voice-response or Web-response system |
| Allocation concealment | Low | Randomization was performed centrally. All patients, investigators, and care providers were aware of the randomized treatment assignments. |
| Blinding of participants and personnel | High | No placebo was used for the standard-therapy group. |
| Blinding of outcome assessment | Low | Potential cardiovascular events were adjudicated by the central clinical-events committee at the TIMI Study Group in Boston, whose members were unaware of treatment assignments. |
| Incomplete outcome data | Low | Serious adverse events occurred in 222 patients (7.5%) in the evolocumab group and in 111 patients (7.5%) in the standard-therapy group. |
| Selective reporting | Low | The primary end point in the two trials was the incidence of adverse events. Serious adverse events occurred in 222 patients (7.5%) in the evolocumab group and in 111 patients (7.5%) in the standard-therapy group. |
| Other bias | Low | No concerns outside the assessed risk of bias domains. |

**Title**: Effect of Evolocumab on Progression of Coronary Disease in Statin-Treated Patients: The GLAGOV Randomized Clinical Trial.

| Year of publication: 2016 | | |
| --- | --- | --- |
| First author: Nicholls SJ | | |
| NCT: NCT01813422 | | |
| **Methods** Type of RCT: A multicenter, double-blind, placebocontrolled, randomized clinical trial Settings: outpatient care  Duration: 76 weeks | | |
| **Participants** Number of participants: 968  Male: 699 (72.2%)  Mean age: 59.8 years  Participants with FH: no data  History of CVD: 628  Inclusion criteria   - Clinical indication for coronary angiography - Subjects already taking statin therapy, niacin or ezetimibe at screening must have been on a stable dose for at least 4 weeks prior to screening LDL-C. Subjects not taking lipid-regulating therapy must enter the study via a lipid stabilization period. Subjects who are intolerant to statins must meet statin intolerance entry criteria - Fasting LDL-C ≥ 80 mg/dL (2.07 mmol/L) with or without additional risk factors, or, LDL-C ≥ 60 -< 80 mg/dL (1.55-2.07 mmol/L) in the presence of one major or three minor risk factors   Exclusion criteria   - Coronary artery bypass graft surgery < 6 weeks prior to the qualifying IVUS - NYHA III or IV heart failure, or last known left ventricular ejection fraction less than 30% - Uncontrolled cardiac arrhythmia that is not controlled by medications in the 3 months prior to randomization - Known hemorrhagic stroke - Uncontrolled hypertension at randomization - Fasting Triglycerides ≥ 400 mg/dL (4.5 mmol/L) at screening - Type 1 diabetes or poorly controlled type 2 diabetes (HbA1c > 9%) at screening. - Moderate to severe renal dysfunction (eGFR < 30 ml/min/1.73m²) at screening. | | |
| **Interventions** Background therapy: statin therapy  Randomized therapy: evolocumab vs placebo  Evolocumab dose: 420 mg every month | | |
| **Primary endpoints**: The primary efficacy end point was the nominal change in percent atheroma volume from baseline to week 78. | | |
| **Outcomes of interest (PCSK9 inhibitor vs control)** VT: 0/484 vs 2/484  VF: 0/484 vs 1/484 | | |
| **Risk of bias** | | |
| **Bias** | **Authors’**  **judgement** | **Support for judgement** |
| Random sequence generation | Low | Used an interactive voice response system |
| Allocation concealment | Low | Patients underwent randomization in a 1:1 allocation  ratio with a block size of 4 using an interactive voice response system to treatment with evolocumab or placebo |
| Blinding of participants and personnel | Low | Using digitized images, personnel unaware of treatment status performed measurements of the lumen and external elastic membrane in images within a matched artery segment. Measurement personnel were blinded to the sequence of imaging studies (baseline vs follow-up). |
| Blinding of outcome assessment | Low | A clinical events committee, blinded to treatment assignment, adjudicated cardiovascular events. |
| Incomplete outcome data | Low | All patients were included in safety analysis. |
| Selective reporting | Low | Reported the predefined endpoints. |
| Other bias | Low | No concerns outside the assessed risk of bias domains. |

**Title**: A phase III randomized trial evaluating alirocumab 300 mg every 4 weeks as monotherapy or add-on to statin: ODYSSEY CHOICE 1.

| Year of publication: 2016 | | |
| --- | --- | --- |
| First author: Roth EM | | |
| NCT: NCT01926782 | | |
| **Methods** Type of RCT: A randomized, double-blind, placebocontrolled, phase 3 multinational study  Settings: outpatient care  Duration: 48 weeks | | |
| **Participants** Number of participants: 803  Male: 462 (57.5%)  Mean age: 60.8 years  Participants with FH: 45 (6%)  History of CVD: No data  Inclusion criteria   - Men and women > age 18 or legal age of majority with elevated LDL-C - Patients not having adequate control of their hypercholesterolemia based on their individual level of CVD risk - Willing and able to comply with clinic visits and study-related procedures - Provided signed informed consent   Exclusion criteria   - Recent (within 3 months prior to the screening visit) myocardial infarction, unstable angina leading to hospitalization, PCI, CABG, uncontrolled cardiac arrhythmia, stroke, transient ischemic attack, carotid revascularization, endovascular procedure or surgical intervention for peripheral vascular disease - Known history of positive test for HIV - Any clinically significant abnormality identified at the time of screening that in the judgment of the investigator or any sub-investigator would preclude safe completion of the study or constrain assessment of endpoints, such as major systemic diseases or participants with short life expectancy. - Participants considered by the investigator or any sub-investigator to be inappropriate for this study (e.g, geographic or social), actual or anticipated, that the investigator felt would restrict or limit the participant's participation for the duration of the study. - Certain laboratory findings obtained during the screening period | | |
| **Interventions** Background therapy: statin therapy  Randomized therapy: alirocumab vs placebo  Alirocumab dose: Alirocumab patients (in the alirocumab 300 mg Q4W and 75 mg Q2W treatment groups) not achieving target LDL-C levels at week 8, or if their LDL-C level was reduced by ≤ 30% from baseline at week 8, had their alirocumab dosing regimen changed to 150mg Q2W at week 12 in a blinded fashion. | | |
| **Primary endpoints**: The co-primary endpoints were the percent change in calculated LDL-C from baseline to Week 24, and the percent change in calculated LDL-C from baseline to the average over Weeks 21-24. | | |
| **Outcomes of interest (PCSK9 inhibitor vs control)** VT: 1/573 vs 0/230 | | |
| **Risk of bias** | | |
| **Bias** | **Authors’**  **judgement** | **Support for judgement** |
| Random sequence generation | Low | Used permuted block design. |
| Allocation concealment | Low | Patients were randomized using a permuted block design. |
| Blinding of participants and personnel | Low | Double-blind treatment |
| Blinding of outcome assessment | Low | This was analyzed separately for the study populations receiving concomitant statin and those not receiving statin. These were analyzed using a validated assay by Regeneron Pharmaceuticals, Inc. |
| Incomplete outcome data | Low | The ITT population included 792 patients overall, and the safety population included 802. |
| Selective reporting | Low | Reported predefined endpoints. |
| Other bias | Low | No concerns outside the assessed risk of bias domains. |

**Title**: Cardiovascular Efficacy and Safety of Bococizumab in High-Risk Patients.

| Year of publication: 2017 | | |
| --- | --- | --- |
| First author: Ridker PM | | |
| NCT: NCT01975376 (SPIRE-1), NCT01975389 (SPIRE-2) | | |
| **Methods** Type of RCT: Phase 3 Multi-center, Double-blind, Randomized, Placebo-controlled, Parallel Group study.  Settings: outpatient care  Duration: Baseline up to 3.4 years. | | |
| **Participants** Number of participants: 16817 (SPIRE-1), 10621 (SPIRE-2)  Male: 12377 (SPIRE-1), 6946 (SPIRE-2)  Mean age: 63.3 years (SPIRE-1), 62.4 years (SPIRE-2)  Participants with FH: 1.8% (SPIRE-1), 7.3% (SPIRE-2)  History of CVD: No data  **SPIRE-1**  Inclusion criteria   - Must be on background lipid lowering treatment. - Must be at high risk of a CV event. - Must have an LDL C ≥ 70 mg/dL (1.8 mmol/L) or non-HDL-C ≥ 100 mg/dL (2.6 mmol/L).   Exclusion criteria   - Planned coronary (PCI or CABG) or other arterial revascularization. - New York Heart Association Class IV congestive heart failure or left ventricular ejection fraction < 25% by cardiac imaging. - Chronic renal insufficiency with creatinine clearance of <30 ml/min/1.73m^2 by MDRD formula or with end state renal disease on dialysis. - History of hemorrhagic stroke. - Prior exposure to bococizumab or other investigational PCSK9 inhibitor.   **SPIRE-2**  Inclusion criteria   - Must be on background lipid lowering treatment. - Must be at high risk of a CV event. - Must have an LDL C ≥ 100 mg/dL (2.6 mmol/L) OR non-HDL C ≥ 130 mg/dL (3.4 mmol/L).   Exclusion criteria   - Planned coronary (PCI or CABG) or other arterial revascularization. - New York Heart Association Class IV congestive heart failure or left ventricular ejection fraction < 25% by cardiac imaging. - Chronic renal insufficiency with creatinine clearance of <30 ml/min/1.73m^2 by MDRD formula or with end state renal disease on dialysis. - History of hemorrhagic stroke. - Prior exposure to bococizumab or other investigational PCSK9 inhibitor. | | |
| **Interventions** Background therapy: statin therapy  Randomized therapy: bococizumab vs placebo  Bococizumab dose: 150mg Q2W | | |
| **Primary endpoints**: The prespecified primary end point of the two trials was a composite of adjudicated and confirmed nonfatal myocardial infarction, nonfatal stroke, hospitalization for unstable angina requiring urgent revascularization, or cardiovascular death. | | |
| **Outcomes of interest (PCSK9 inhibitor vs control)** **SPIRE-1**  VT: 4/8408 vs 9/8409  VF: 3/8408 vs 0/8409  SCD: 3/8408 vs 1/8409  Cardiac arrest: 8/8408 vs 3/8409  **SPIRE-2**  VT: 5/5312 vs 5/5309  VF: 1/5312 vs 0/5309  SCD: 5/5312 vs 8/5309  Cardiac arrest: 4/5312 vs 5/5309 | | |
| **Risk of bias** | | |
| **Bias** | **Authors’**  **judgement** | **Support for judgement** |
| Random sequence generation | Low | Used computer generated randomization schedule. |
| Allocation concealment | Low | During the treatment period, interactive response technology interactions will be used for the allocation of investigational product and to monitor treatments status. |
| Blinding of participants and personnel | Low | The study will be subject, investigator, and sponsor blinded. |
| Blinding of outcome assessment | Low | All potential clinical endpoints are collected for all randomized subjects through study completion/end of study visit whether or not the subject is on double-blind investigational product. |
| Incomplete outcome data | Low | AEs should be recorded on the CRF from the time the subject has signed the informed consent document through last subject visit. |
| Selective reporting | Low | All observed or volunteered AEs regardless of treatment group or suspected causal relationship to the investigational products will be reported. |
| Other bias | Low | No concerns outside the assessed risk of bias domains. |

**Title**: Lipid-Reduction Variability and Antidrug-Antibody Formation with Bococizumab.

| Year of publication: 2017 | | |
| --- | --- | --- |
| First author: Ridker PM | | |
| NCT: NCT01968967 (SPIRE-LDL), NCT02100514 (SPIRE-LL) | | |
| **Methods** Type of RCT: A multicenter, randomized study  Settings: outpatient care  Duration: Participants received placebo matched to Bococizumab subcutaneous injection once every 2 weeks up to Week 52. Participants were followed up to Week 58. | | |
| **Participants** Number of participants: 2139 (SPIRE-LDL), 746 (SPIRE-LL)  Male: 1270 (SPIRE-LDL), 416 (SPIRE-LL)  Mean age: 62.0 years (SPIRE-LDL), 61.6 years (SPIRE-LL)  Participants with FH: 1.9% (SPIRE-LDL), 7.0% (SPIRE-LL)  History of CVD: No data  **SPIRE-LDL**  Inclusion criteria   - Treated with a statin. - Fasting LDL-C > 70 mg/dL and triglyceride <=400 mg/dL. - High or very high risk of incurring a cardiovascular event.   Exclusion criteria   - Pregnant or breastfeeding females. - Cardiovascular or cerebrovascular event of procedures during the past 30 days. - Congestive heart failure NYHA class IV. - Poorly controlled hypertension.   **SPIRE-LL**  Inclusion criteria   - Treated with a statin - Fasting LDL-C >=100 mg/dL and triglyceride <= 400 mg/dL - High or very high risk of incurring a cardiovascular event   Exclusion criteria   - Pregnant or breastfeeding females - Cardiovascular or cerebrovascular event or procedure within 90 days - Congestive heart failure NYHA class IV - Poorly controlled hypertension | | |
| **Interventions** Background therapy: statin therapy  Randomized therapy: bococizumab vs placebo  Bococizumab dose: 150mg Q2W | | |
| **Primary endpoints**: The primary end point was the percent change from baseline in fasting LDL cholesterol levels, as measured with a direct assay at week 12. | | |
| **Outcomes of interest (PCSK9 inhibitor vs control)** **SPIRE-LDL**  VT: 2/1068 vs 1/1071  Cardiac arrest: 2/1068 vs 0/1071  **SPIRE-LL**  VT: 1/499 vs 0 /247 | | |
| **Risk of bias** (The protocols for each SPIRE study were collaboratively designed by academic members of the SPIRE executive and steering committees and physician and statistician employees of the sponsor.) | | |
| **Bias** | **Authors’**  **judgement** | **Support for judgement** |
| Random sequence generation | Low | Used computer generated randomization schedule. |
| Allocation concealment | Low | This is a double-blind, parallel group study. Subjects will be stratified by TG level and geographic region. |
| Blinding of participants and personnel | Low | The study will be subject, investigator, and sponsor blinded. |
| Blinding of outcome assessment | Low | The study will be subject, investigator, and sponsor blinded. ECGs may be performed for the evaluation of adverse events at the discretion of the investigator. |
| Incomplete outcome data | Low | Per protocol, we examined safety events and the incidence of adjudicated cardiovascular events occurring after the time of randomization. All AEs will be recorded in the AE CRF from the time the subject signs the informed consent to the end of study visit. |
| Selective reporting | Low | All AEs will be recorded in the AE CRF from the time the subject signs the informed consent to the end of study visit. For all AEs, sufficient information should be obtained by the investigator to determine the causality of the AE. |
| Other bias | Low | No concerns outside the assessed risk of bias domains. |

**Title**: Evolocumab and Clinical Outcomes in Patients with Cardiovascular Disease. (FOURIER)

| Year of publication: 2017 | | |
| --- | --- | --- |
| First author: Sabatine MS | | |
| NCT: NCT01764633 | | |
| **Methods** Type of RCT: A randomized, doubleblind, placebo-controlled, multinational clinical trial  Settings: outpatient care  Duration: 157 weeks | | |
| **Participants** Number of participants: 27564  Male: 20795 (75.5%)  Mean age: 62.5 years  Participants with FH: no data  History of CVD: All participants  Inclusion criteria   - Male or female ≥ 40 to ≤ 85 years of age - History of clinically evident cardiovascular disease at high risk for a recurrent event - Fasting low-density lipoprotein cholesterol (LDL-C) ≥ 70 mg/dL (≥ 1.8 mmol/L) ) or non-high-density lipoprotein cholesterol (non-HDL-C) ≥ 100 mg/dL (> 2.6 mmol/L) - Fasting triglycerides ≤ 400 mg/dL (4.5 mmol/L)   Exclusion criteria   - NYHA class III or IV, or last known left ventricular ejection fraction < 30% - Uncontrolled hypertension - Uncontrolled or recurrent ventricular tachycardia - Untreated hyperthyroidism or hypothyroidism - Homozygous familial hypercholesterolemia - LDL or plasma apheresis | | |
| **Interventions** Background therapy: statin therapy  Randomized therapy: evolocumab vs placebo  Evolocumab dose: either 140 mg every 2 weeks or 420 mg every month, according to patient preference | | |
| **Primary endpoints**: The primary efficacy end point was major cardiovascular events, defined as the composite of cardiovascular death, MI, stroke, hospitalization for unstable angina, or coronary revascularization. | | |
| **Outcomes of interest (PCSK9 inhibitor vs control)** VT: 25/13784 vs 27/13780  VF: 12/13784 vs 8/13780  SCD: 0/13784 vs 1/13780  Cardiac arrest: 3/13784 vs 5/13780 | | |
| **Risk of bias** | | |
| **Bias** | **Authors’**  **judgement** | **Support for judgement** |
| Random sequence generation | Low | Central computerized system. |
| Allocation concealment | Low | Central allocation. |
| Blinding of participants and personnel | Low | Both were blinded. |
| Blinding of outcome assessment | Low | Central laboratory and blinded adjudication. |
| Incomplete outcome data | Low | A total of 27525 patients (99.9%) received at least one dose of a study agent. Ascertainment of the primary end point was complete for 99.5% of potential patient-years of follow-up. |
| Selective reporting | Low | Reported most endpoints. |
| Other bias | Low | No concerns outside the assessed risk of bias domains. |

**Title**: Alirocumab and Cardiovascular Outcomes after Acute Coronary Syndrome. (DOYSSEY OUTCOMES)

| Year of publication: 2018 | | |
| --- | --- | --- |
| First author: Schwartz GG | | |
| NCT: NCT01663402 | | |
| **Methods** Type of RCT: A multicenter, randomized, double-blind, placebo-controlled trial.  Settings: Outpatient care  Duration: 2.8 years | | |
| **Participants** Number of participants: 18924  Male: 14162 (74.8%)  Mean age: 58.6 years  Participants with FH: No data  History of CVD: No  Inclusion criteria: Recently (< 52 weeks) hospitalized for ACS.  Exclusion criteria   - Age < 40 years. - ACS event occurring more than 52 weeks prior to randomization visit. - LDL-C likely to be <70 mg/dL (<1.81 mmo/L), and apolipoprotein B (ApoB) <80 mg/dL (<0.8 g/L), and non - high-density lipoprotein cholesterol (HDL-C) <100 mg/dL (<2.59 mmol/L) with evidence-based medical and dietary management of dyslipidemia. | | |
| **Interventions** Background therapy: Maximum tolerated dose of one of these statins.  Randomized therapy: alirocumab vs placebo  Alirocumab dose: Alirocumab 75 mg SC injection Q2W added to stable LMT for up to 64 months. | | |
| **Primary endpoints**: The primary end point was a composite of death from coronary heart disease, nonfatal myocardial infarction, fatal or nonfatal ischemic stroke, or unstable angina requiring hospitalization. | | |
| **Outcomes of interest (PCSK9 inhibitor vs control)** VT: 24/9462 vs 29/9462  VF: 8/9462 vs 17/9462  SCD: 36/9462 vs 42/9462  Cardiac arrest: 9/9462 vs 9/9462 | | |
| **Risk of bias** | | |
| **Bias** | **Authors’**  **judgement** | **Support for judgement** |
| Random sequence generation | Low | Two types of centralized treatment allocation system will be used, the Interactive Voice Response System (IVRS) and the Interactive Web Response System (IWRS) depending on the choice of the site. |
| Allocation concealment | Low | Before randomizing a patient, the Investigator or designee will have to contact the centralized treatment allocation system. |
| Blinding of participants and personnel | Low | The double-blind treatment period will continue until each surviving randomized patient has been followed for a minimum of 24 months or the target number of events is reached, whichever comes last. |
| Blinding of outcome assessment | Low | An independent data and safety monitoring committee monitored the safety and efficacy data. All primary and secondary end points were adjudicated by physicians who were unaware of the trial-group assignments. |
| Incomplete outcome data | Low | Analyses were performed according to the intention-to-treat principle and included data from all patients and for all events that occurred from the time of randomization to the common trial end date. |
| Selective reporting | Low | All primary and secondary end points were adjudicated by physicians who were unaware of the trial-group assignments. The protocol specified that the trial was to continue until at least 1613 primary end-point events had occurred and all patients who could be evaluated were followed for at least 2 years, which would ensure a sufficient observation time in which to assess safety and efficacy. |
| Other bias | Low | No concerns outside the assessed risk of bias domains. |

**Title**: Inclisiran for the Treatment of Heterozygous Familial Hypercholesterolemia. (ORION-9)

| Year of publication: 2020 | | |
| --- | --- | --- |
| First author: Raal FJ | | |
| NCT: NCT03397121 | | |
| **Methods** Type of RCT: A double-blind, randomized, placebo-controlled trial.  Settings: Outpatient care  Duration: 510 days | | |
| **Participants** Number of participants: 482  Male: 227 (47.1%)  Mean age: 54.7 years  Participants with FH: All.  History of CVD: 132  Inclusion criteria   - Male or female participants ≥18 years of age. - History of HeFH with a diagnosis of HeFH by genetic testing; and/or a documented history of untreated LDL-C of >190 mg/dL, and a family history of familial hypercholesterolemia, elevated cholesterol or early heart disease that may indicate familial hypercholesterolemia. - Serum LDL-C ≥2.6 millimoles (mmol)/liter (L) (≥100 mg/dL) at screening. - Fasting triglyceride <4.52 mmol/L (<400 mg/dL) at screening. - Participants on statins should be receiving a maximally tolerated dose. - Participants not receiving statins must have documented evidence of intolerance to all doses of at least 2 different statins. - Participants on lipid-lowering therapies (such as a statin and/or ezetimibe) should be on a stable dose for ≥30 days before screening with no planned medication or dose change during study participation.   Exclusion criteria   - New York Heart Association (NYHA) class IV heart failure. - Uncontrolled cardiac arrhythmia - Uncontrolled severe hypertension - Active liver disease - Females who are pregnant or nursing, or who are of childbearing potential and unwilling to use at least 2 methods of highly effective contraception (failure rate less than 1% per year) (combined oral contraceptives, barrier methods, approved contraceptive implant, long-term injectable contraception, or intrauterine device) for the entire duration of the study. Exemptions from this criterion: 1) Women >2 years postmenopausal (defined as 1 year or longer since last menstrual period) AND more than 55 years of age. 2) Postmenopausal women (as defined above) and less than 55 years of age with a negative pregnancy test within 24 hours of randomization. 3) Women who are surgically sterilized at least 3 months prior to enrollment. - Males who are unwilling to use an acceptable method of birth control during the entire study period (condom with spermicide). - Treatment with other investigational products or devices within 30 days or 5 half-lives of the screening visit, whichever is longer. - Treatment (within 90 days of screening) with monoclonal antibodies directed towards PCSK9. | | |
| **Interventions** Background therapy: A maximally accepted dose of statin therapy with or without ezetimibe.  Randomized therapy: inclisiran vs placebo  Inclisiran dose: Inclisiran sodium 1.5-ml (equivalent to 284 mg inclisiran) subcutaneous injection on days 1, 90, 270, and 450. | | |
| **Primary endpoints**: The two primary end points were the percent change from baseline in the LDL cholesterol level at day 510 and the time-adjusted percent change from baseline in the LDL cholesterol level between day 90 and day 540. | | |
| **Outcomes of interest (PCSK9 inhibitor vs control)** Cardiac arrest: 1/242 vs 0/240 | | |
| **Risk of bias** | | |
| **Bias** | **Authors’**  **judgement** | **Support for judgement** |
| Random sequence generation | Low | Randomization via automated interactive response technology (IRT) will be used to assign subject to blinded investigational product kits. |
| Allocation concealment | Low | This is a double-blind placebo-controlled study. Study medication will be blinded prior to distribution to the site. Randomization via an automated IRT will be used to assign subjects to blinded investigational product. |
| Blinding of participants and personnel | Low | This is a double-blind placebo-controlled study. |
| Blinding of outcome assessment | Low | All SAEs that occur during the designated study period from consent through EOS must be reported to MDCO GPV Department within 24 hours of awareness of the event. |
| Incomplete outcome data | Low | Adverse events and laboratory values were recorded at all visits through the end-of-trial visit on day 540. Adverse events that occurred during the trial period, regardless of causality, were reported in 185 of 241 patients (76.8%) in the inclisiran group and in 172 of 240 patients (71.7%) in the placebo group. |
| Selective reporting | Low | All SAEs that occur during the designated study period from consent through EOS must be reported to MDCO GPV Department within 24 hours of awareness of the event. |
| Other bias | Low | No concerns outside the assessed risk of bias domains. |

**Title**: Two Phase 3 Trials of Inclisiran in Patients with Elevated LDL Cholesterol.

| Year of publication: 2020 | | |
| --- | --- | --- |
| First author: Ray KK | | |
| NCT: NCT03399370 (ORION-10), NCT03400800 (ORION-11) | | |
| **Methods** Type of RCT: two randomized, double-blind, placebo-controlled, parallel-group, phase 3 trials.  Settings: outpatient care  Duration: 540 days | | |
| **Participants** Number of participants: 1561 (ORION-10), 1617 (ORION-11)  Male: 1083 (ORION-10), 1160 (ORION-11)  Mean age: 66.1 years (ORION-10), 64.8 years (ORION-11)  Participants with FH: 20 (ORION-10), 28 (ORION-11)  History of CVD: 1561 (ORION-10), 1414 (ORION-11)  **ORION-10**  Inclusion criteria   - Male or female participants ≥18 years of age. - History of ASCVD (CHD, CVD, or PAD). - Serum LDL-C ≥1.8 millimole (mmol)/liter (L) (≥70 mg/dL). - Fasting triglyceride <4.52 mmol/L (<400 mg/dL) at screening. - Participants on statins should be receiving a maximally tolerated dose. - Participants not receiving statins must have documented evidence of intolerance to all doses of at least 2 different statins. - Subjects on lipid-lower therapies (such as a statin and/or ezetimibe) should be on a stable dose for ≥30 days before screening with no planned medication or dose change during study participation.   Exclusion criteria   - New York Heart Association (NYHA) class IV heart failure. - Uncontrolled cardiac arrhythmia - Uncontrolled severe hypertension - Active liver disease - Females who are pregnant or nursing, or who are of childbearing potential and unwilling to use at least 2 methods of highly effective contraception (failure rate less than 1% per year) (for example, combined oral contraceptives, barrier methods, approved contraceptive implant, long-term injectable contraception, or intrauterine device) for the entire duration of the study. Exemptions from this criterion: 1) Women >2 years postmenopausal (defined as 1 year or longer since last menstrual period) and more than 55 years of age. 2) Postmenopausal women (as defined above) and less than 55 years of age with a negative pregnancy test within 24 hours of randomization. 3) Women who are surgically sterilized at least 3 months prior to enrollment. - Males who are unwilling to use an acceptable method of birth control during the entire study period (such as condom with spermicide). - Treatment with other investigational products or devices within 30 days or 5 half-lives of the screening visit, whichever is longer. - Treatment (within 90 days of screening) with monoclonal antibodies directed towards PCSK9   **ORION-11**  Inclusion criteria   - Male or female participants ≥18 years of age. - History of ASCVD (CHD, CVD, or PAD). - Serum LDL-C ≥1.8 millimole (mmol)/liter (L) (≥70 mg/dL). - Fasting triglyceride <4.52 mmol/L (<400 mg/dL) at screening. - Calculated glomerular filtration rate >30 mL/min by eGFR using standardized clinical methodology - Participants on statins should be receiving a maximally tolerated dose. - Participants not receiving statins must have documented evidence of intolerance to all doses of at least 2 different statins. - Subjects on lipid-lower therapies (such as a statin and/or ezetimibe) should be on a stable dose for ≥30 days before screening with no planned medication or dose change during study participation. - Subjects were willing and able to give informed consent before initiation of any study-related procedures and willing to comply with all required study procedures   Exclusion criteria   - New York Heart Association (NYHA) class IV heart failure. - Uncontrolled cardiac arrhythmia - Uncontrolled severe hypertension - Active liver disease - Females who are pregnant or nursing, or who are of childbearing potential and unwilling to use at least 2 methods of highly effective contraception (failure rate less than 1% per year) (for example, combined oral contraceptives, barrier methods, approved contraceptive implant, long-term injectable contraception, or intrauterine device) for the entire duration of the study. Exemptions from this criterion: 1) Women >2 years postmenopausal (defined as 1 year or longer since last menstrual period) and more than 55 years of age. 2) Postmenopausal women (as defined above) and less than 55 years of age with a negative pregnancy test within 24 hours of randomization. 3) Women who are surgically sterilized at least 3 months prior to enrollment. - Males who are unwilling to use an acceptable method of birth control during the entire study period (such as condom with spermicide). - Treatment with other investigational products or devices within 30 days or 5 half-lives of the screening visit, whichever is longer. - Treatment (within 90 days of screening) with monoclonal antibodies directed towards PCSK9 | | |
| **Interventions** Background therapy: statin therapy and/or ezetimibe  Randomized therapy: inclisiran vs placebo  Inclisiran dose: Inclisiran sodium 1.5-ml (equivalent to 284 mg inclisiran) subcutaneous injection on days 1, 90, 270, and 450. | | |
| **Primary endpoints**: The coprimary end points in each trial were the placebo-corrected percentage change in LDL cholesterol level from baseline to day 510 and the time-adjusted percentage change in LDL cholesterol level from baseline after day 90 and up to day 540. | | |
| **Outcomes of interest (PCSK9 inhibitor vs control)** **ORION-10**  VT: 2/781 vs 4/780  VF: 0/781 vs 2/780  Cardiac arrest: 1/781 vs 1/780  **ORION-11**  VT: 1/810 vs 1/807  VF: 1/810 vs 0/807  Cardiac arrest: 3/810 vs 0/807 | | |
| **Risk of bias** | | |
| **Bias** | **Authors’**  **judgement** | **Support for judgement** |
| Random sequence generation | Low | Randomization via automated interactive response technology. |
| Allocation concealment | Low | Investigational product will be dispensed and administered in a blinded syringe. |
| Blinding of participants and personnel | Low | This is a double-blind placebo-controlled study. |
| Blinding of outcome assessment | Low | Subjects will be carefully monitored for adverse events by the investigator during the designated study period. |
| Incomplete outcome data | Low | In the ORION-10 trial, the safety population comprises 781 patients in the inclisiran group and 778 patients in the placebo group. In the ORION-11 trial, the safety population of the latter trial comprises 811 patients exposed to inclisiran and 804 patients exposed to placebo. |
| Selective reporting | Low | Adverse events that occurred during the trial period, regardless of causality, were reported in 574 of 781 patients (73.5%) receiving inclisiran and 582 of 778 (74.8%) receiving placebo in the ORION-10 trial and in 671 of 811 patients (82.7%) receiving inclisiran and 655 of 804 (81.5%) receiving placebo in the ORION-11 trial. |
| Other bias | Low | No concerns outside the assessed risk of bias domains. |
