# Supplementary material for: Evaluating the potential effect of PCSK9 inhibitors on the risk of sudden cardiac death and ventricular arrhythmias: A meta-analysis of randomized controlled trials
Source: PLoS One. 2025 Aug 8;20(8):e0329676. doi: 10.1371/journal.pone.0329676 (PMC12334025; doi:10.1371/journal.pone.0329676)
Supplement: S5 Table — (DOCX) [file pone.0329676.s005.docx]

**S5 Table. GRADE certainty of evidence**

| **Certainty assessment** | | | | | | | **No of patients** | | **Effect** | | **Quality** |
| --- | --- | --- | --- | --- | --- | --- | --- | --- | --- | --- | --- |
| **No of**  **studies** | **Study design** | **Risk of bias** | **Inconsistency** | **Indirectness** | **Imprecision** | **Other consideration** | **PCSK9 inhibitors** | **Control** | **Odds ratio** | **95% CI** |  |
| Sudden cardiac death | | | | | | | | | | | |
| 3 | Randomized  trials | No serious | No serious | Serious | Serious | None | 37/23725  (1.4‰) | 44/23484  （1.9‰） | 0.83 | 0.54-1.29 | ⊕⊕〇〇  Low |
| Ventricular arrhythmias | | | | | | | | | | | |
| 11 | Randomized  trials | No serious | No serious | Serious | Serious | None | 80/31586  (2.5‰) | 95/28398  (3.3‰) | 0.81 | 0.60-1.09 | ⊕⊕〇〇  Low |
| Cardiac arrest | | | | | | | | | | | |
| 9 | Randomized  trials | No serious | No serious | Serious | Serious | None | 24/30433  (0.7‰) | 15/27743  (0.5‰) | 1.20 | 0.61-2.34 | ⊕⊕〇〇  Low |
