# Supplementary material for: Evaluating the potential effect of PCSK9 inhibitors on the risk of sudden cardiac death and ventricular arrhythmias: A meta-analysis of randomized controlled trials
Source: PLoS One. 2025 Aug 8;20(8):e0329676. doi: 10.1371/journal.pone.0329676 (PMC12334025; doi:10.1371/journal.pone.0329676)
Supplement: S1 File — (DOCX) [file pone.0329676.s006.docx]

**Sensitivity analysis based on reviewer’s comments.**

Sudden cardiac death (SCD)

Three trials reported outcomes of SCD. One trial reported zero-event in the PCSK9 inhibitor group. Excluding this trial, the overall effect trend showed no substantial changes (RR 0.85, 95% CI 0.54-1.31; P=0.71; *I*^2^=0%).

Ventricular arrhythmias

Fifteen trials reported outcomes of ventricular arrhythmias. One trial found no events in both arms, and five reported no events in a single arm. Excluding the trial with no events in both arms, PCSK9 inhibitors therapy did not significantly reduce the risk of ventricular arrhythmias (RR 0.81, 95% CI 0.60-1.09; P=0.17; *I*^2^=0%). Similarly, excluding trials with no events in a single arm showed no substantial change in the overall effect (RR 0.82, 95% CI 0.60-1.11; P=0.44; *I*^2^=0%).
