# Supplementary material for: Evaluating the potential effect of PCSK9 inhibitors on the risk of sudden cardiac death and ventricular arrhythmias: A meta-analysis of randomized controlled trials
Source: PLoS One. 2025 Aug 8;20(8):e0329676. doi: 10.1371/journal.pone.0329676 (PMC12334025; doi:10.1371/journal.pone.0329676)
Supplement: S2 File — (DOCX) [file pone.0329676.s007.docx]

**Figures of meta-analyses using the Bayesian method**


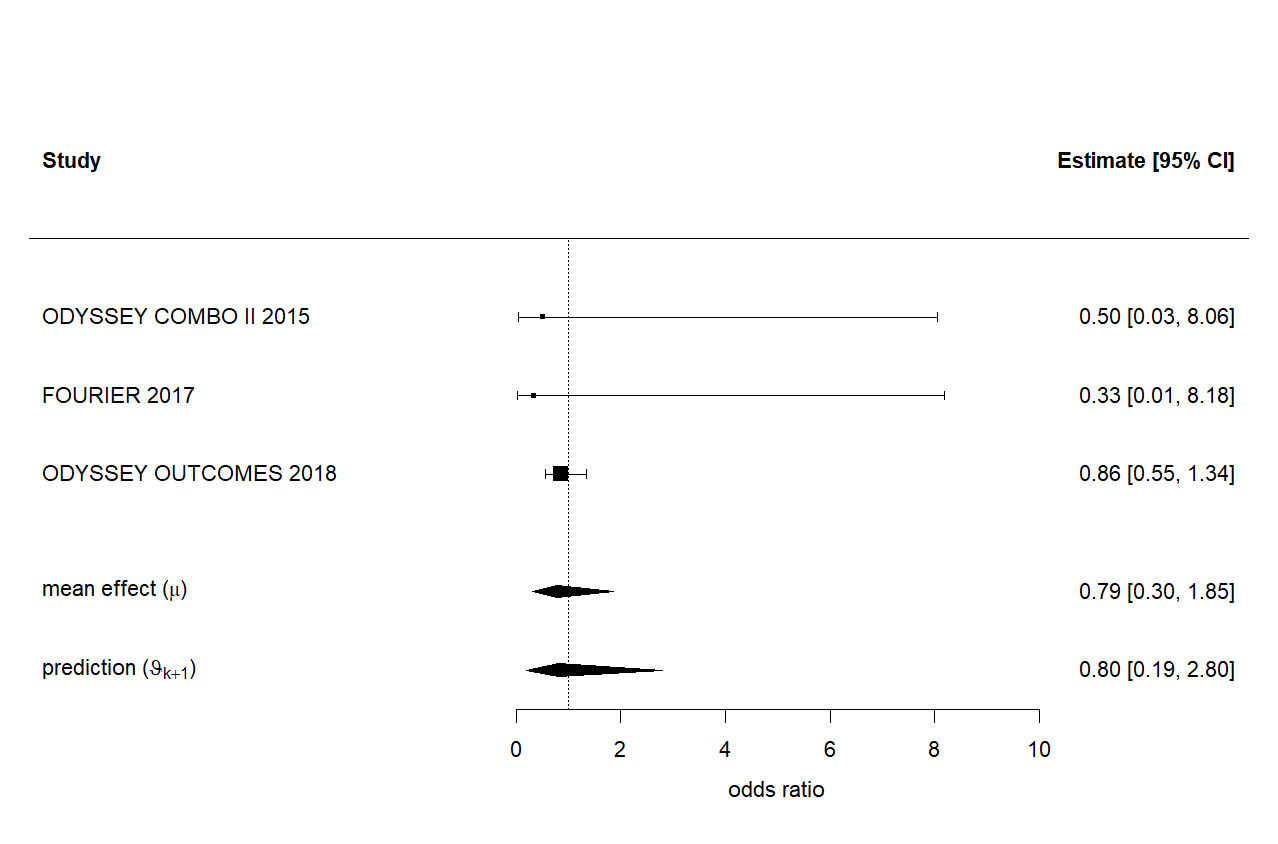


**A**

Fig A. Sudden cardiac death.


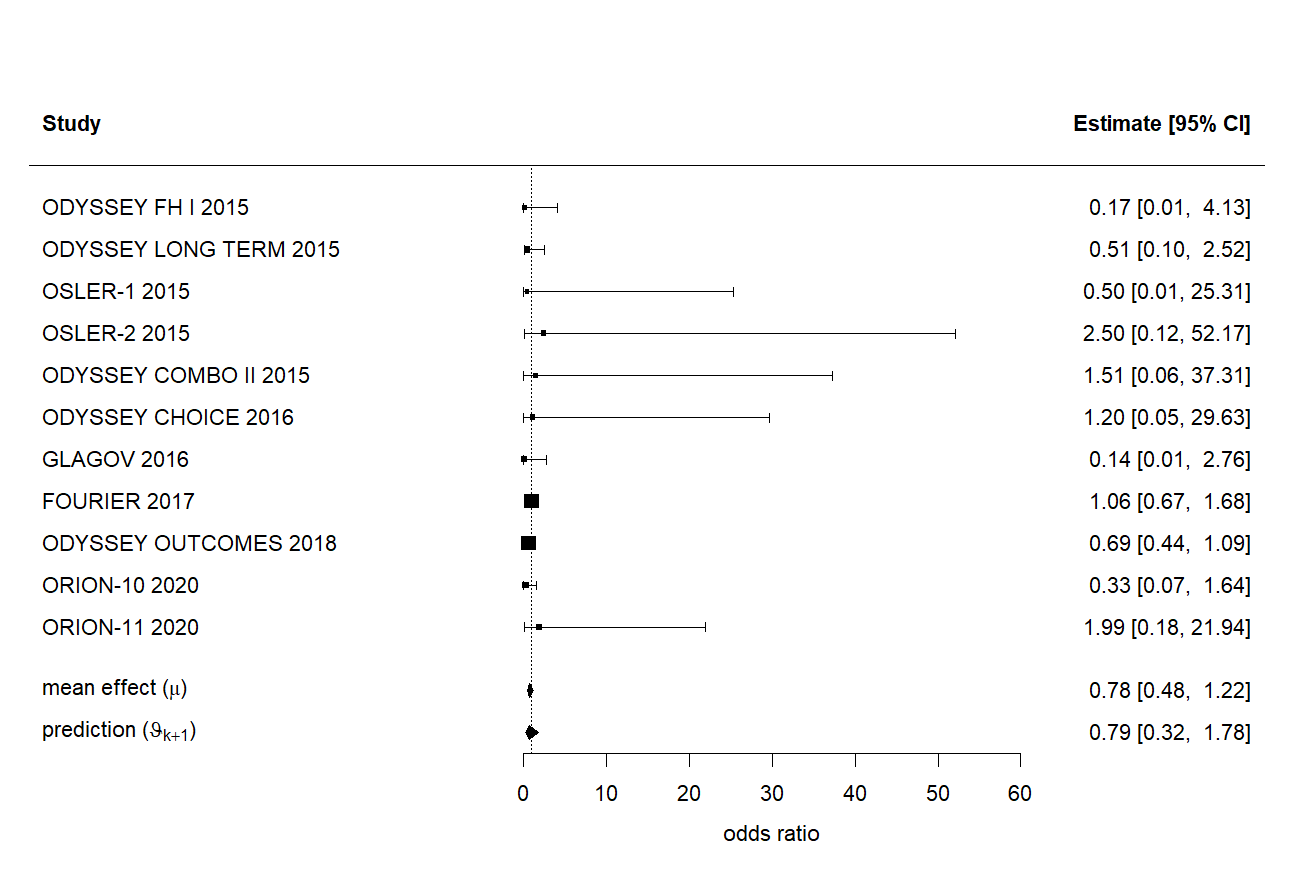


**B**

Fig B. Ventricular arrhythmias.
